# Retraction: Large intergenic non-coding RNA-ROR reverses gemcitabine-induced autophagy and apoptosis in breast cancer cells

**DOI:** 10.18632/oncotarget.28787

**Published:** 2025-12-31

**Authors:** Yao-Min Chen, Yu Liu, Hai-Yan Wei, Ke-Zhen Lv, Pei-Fen Fu

**Affiliations:** ^1^Department of Breast Surgery, The First Affiliated Hospital of Zhejiang University, Hangzhou 310000, P.R. China

**This article has been retracted:** Oncotarget has completed its investigation of this article and found several image duplications. Specifically, Figure 3A contains a scatter plot also used in the earlier published paper Figure 4 [1], which has since been retracted. This plot also appears in Figures of [2, 3], published concurrently, both of which have also been retracted. And the same plots appeared in later published papers [4–8]. We also found internal duplication in Figure 7B, where TEM ultrastructure image of MDA-MB-231 cells treated with “Gem” is highly similar to the image for cells treated with “shCtrl+Gem” and identical to the image of Gem-treated cells in Figure 2B. Additionally, the fluorescent image for cells treated with “Gem” in Figure 2A overlaps with the “Blank” image in Figure 7A and two other images in Figure 7A (shCtrl+Gem and shROR+Gem) also overlap. In light of these findings, the Editorial decision was made to retract this paper.

Oncotarget has contacted both the authors and members of the faculty of their institution (The First Affiliated Hospital of Zhejiang University, Hangzhou, China), regarding these issues, but we have received no response to date.

Original article: Oncotarget. 2016; 7:59604–59617. 59604-59617. https://doi.org/10.18632/oncotarget.10730
